# Value of PET-CT in plasma cell dyscrasias: a literature and pictorial review

**DOI:** 10.1186/1470-7330-15-S1-P11

**Published:** 2015-10-02

**Authors:** R Aggarwal, Y Griffin

**Affiliations:** 1 Leicester Royal Infirmary, Infirmary Square, Leicester, LE1 5WW, UK

## Learning objectives

• To evaluate indications for PET-CT in plasma cell dyscrasias and its added value over other imaging modalities with review of literature based evidence and reference to the RCR/RCP guidelines.

• To discuss the use of PET-CT and subsequent clinical impact at our institution with pictorial illustration.

• To discuss the limitations and potential pitfalls of PET-CT with pictorial illustration.

## Content organisation

• Role of imaging in the diagnosis, management and follow up of plasma cell dyscrasias.

• Indications for PET-CT and circumstances in which it is and is not likely to be beneficial with review of literature based evidence and RCR/RCP guidelines.

• Use of PET-CT at our institution with subsequent clinical impact in staging of non-secretory disease, myeloma, POEMS disease, assessing plasmacytoma response to treatment and MGUS transformation to myeloma with pictorial illustration.

• Limitations and potential pitfalls of PET-CT with pictorial illustration.

## Conclusion

Although PET-CT is recommended by Durie-Salmon Plus, it is not widely adopted. RCR guidelines advise PET-CT for monitoring non secretory myeloma and assessing active disease. At our institution, PET-CT influenced patient management in 95%. PET-CT is useful in staging myeloma, in detection of occult bone/nodal disease and in detecting residual active disease or recurrent disease post chemoradiotherapy/bone marrow transplant. It is of less value in diffuse bone marrow involvement. PET-CT has added value to conventional imaging techniques especially when they are normal, indeterminate or contraindicated.

